# Comprehensive Power Gain Assessment of GaN-SOI-FinFET for Improved RF/Wireless Performance Using TCAD

**DOI:** 10.3390/mi13091418

**Published:** 2022-08-28

**Authors:** Ajay Kumar, Neha Gupta, Amit Kumar Goyal, Yehia Massoud

**Affiliations:** 1 ECE Department, Jaypee Institute of Information Technology, Noida 201309, India; 2 ASH Department, Dr. Akhilesh Das Gupta Institute of Technology & Management, New Delhi 110053, India; 3 Innovative Technologies Laboratories (ITL), King Abdullah University of Science and Technology (KAUST), Thuwal 23955, Saudi Arabia

**Keywords:** cut-off frequency, GaN-SOI-FinFET, maximum oscillator frequency, power gains, RF

## Abstract

In this work, we present a radio frequency (RF) assessment of the nanoscale gallium nitride-silicon-on-insulator fin field-effect transistor (GaN-SOI-FinFET). All the performances of the device were compared with GaN-FinFET and conventional FinFET (Conv. FinFET) simultaneously. All the results show that the power gains significantly improved in terms of Gma, Gms, Stern stability factor (SS), GMT, and intrinsic delay in comparison with conventional FinFET. Current gain and unilateral power gain were also evaluated for the extraction of fT (cut-off frequency) and *f*_MAX_, respectively. *f*_T_ and *f*_MAX_ were enhanced by 88.8% and 94.6%, respectively. This analysis was performed at several THz frequencies. Further, the parametric assessment was also performed in terms of gate length and oxide thickness to find the optimized value of gate length and oxide thickness. The implementation of GaN in the channel reduces the parasitic capacitance and paves the way for high-performance RF applications.

## 1. Introduction

Continuous scaling of metal oxide semiconductor (MOS) devices is required for high speed, better performance, and higher power efficiency. Scaling leads to undesirable short channel effects (SCEs) and parasitic capacitances in an MOS device, which make it unsuitable for RF application. To overcome these effects, 3D structures based on silicon, such as FinFETs [1,2,3], gate-all-around FinFET and FETs [4,5,6], and many FETs with multi-gate, have been developed [7,8]. In recent years, different materials have been introduced in nanochannel transistors, which exhibit better performance. Such devices present near ideal subthreshold slope and lower leakage currents [9]. Gallium nitride (GaN) is used for high voltage and frequency operation owing to its larger bandgap, higher electron mobility, and ability to operate at very high temperatures without degrading its characteristics [10,11]. The FinFETs based on GaN have better gate controllability due to the nonplanar 3D structure [12]. Very low thermal resistance has been observed in GaN material, which makes it suitable for power transistors [13]. Therefore, the self-heating effect was not considered in this analysis. Many other properties make it a more desirable semiconductor for high frequency (THz range) applications [14,15].

This work emphasizes the RF performance of GaN-SOI-FinFET and compares it with two other devices called GaN-FinFET and Conv. FinFET using TCAD. Many figures of merit, such as Gma, Gms, Stern stability factor (SS), G_MT_, and intrinsic delay, were considered and compared with their counterparts. Current gain and unilateral power gain were also considered for the extraction of *f*_T_ and *f*_MAX_. Further, parametric assessment was also performed in terms of gate length and oxide thickness to find the optimized value of gate length and oxide thickness. Due to the rising need for high-speed electronics goods, precise modelling of a FinFET device is needed at high frequencies to demonstrate the behaviours in microwave circuits.

## 2. Device Structure and Simulation Methodology

**Figure 1:**
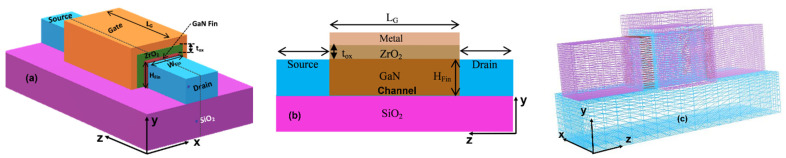
(**a**) Three-dimensional Device structure of GaN-SOI-FinFET, (**b**) 2D view of GaN-SOI-FinFET, and (**c**) 3D meshed device structure.

Figure 1 shows the three-dimensional and two-dimensional device design of the GaN-SOI-FinFET. The proposed device structure (shown in Figure 1) is heterojunction-free; there is no 2DEG (2D electron gas). For this work, 8 nm gate length (L_G_) was considered along with the oxide thickness (t_ox_) of 1 nm on all three fins’ sides. The width (W_Fin_) and height (H_Fin_) of the fin are 4 nm and 8 nm, respectively. Low doping concentration (10^16^ cm^−3^) was considered in the channel region (N_Ch_) uniformly; however, high (10^21^ cm^−3^) doping concentration of Si (uniform) was considered in the source/drain (N_S,D_) regions The device parameters are summarized in Table 1. ZrO_2_ is used as high-k material to reduce leakage currents and increase the physical oxide thickness without significantly increasing the actual effective oxide thickness of gate dielectrics. The increased physical thickness contributes to the reduction in tunneling of carriers through the dielectric. The gate electrode work function is 5.0 eV (nickel). 

In this work, the proposed device (GaN-SOI-FinFET) was simultaneously compared with GaN-FinFET and conventional FinFET. GaN-FinFET architecture has the same dimensions as the proposed device without SOI and high-k material (ZrO_2_). Conventional FinFET has silicon (Si) in the channel region instead of GaN. In the conventional device, high-k material (ZrO_2_) was also not used, and the rest of the device dimensions are the same as the proposed device.

We used a TCAD device simulator to perform the entire simulation [16]. We performed the numerical simulation followed by device design in this work. The construction of structure and numerical resolution are the two key processes in TCAD device simulation. While the numerical element entails defining the work function and selecting the necessary physical models and mathematical techniques to be employed in the simulation, structure design entails establishing the mesh, then specifying the various regions in the device, the doping, and the electrodes [16]. The constant voltage and temperature model was considered for the numerical simulation of the device and this model is used to account for the mobility degradation and the ionized impurity scattering caused due to surface roughness scattering, photon scattering, and field-dependent mobility. Next, we considered the Shockley Read Hall model for recombination effects, which is useful for the leakage current simulation.

## 3. Calibration with Experimental Data

**Figure 2:**
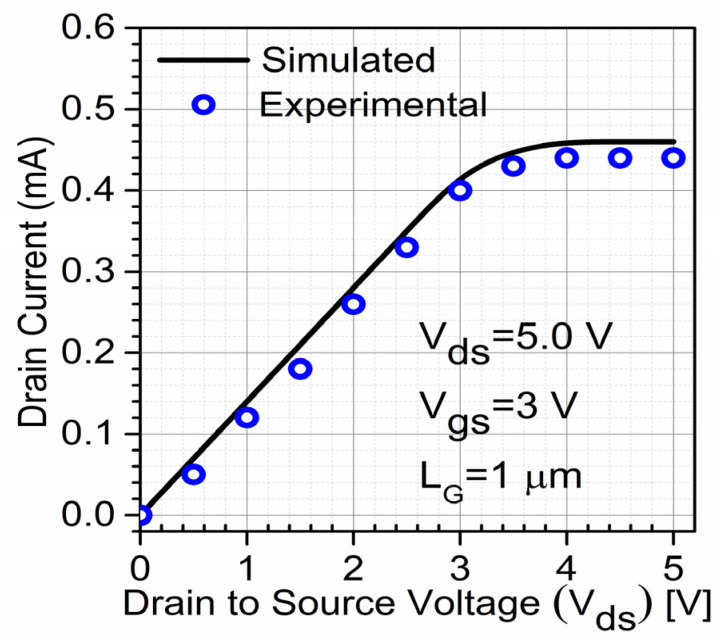
I_ds_-V_ds_ characteristics calibration of experimental GaN FinFET [17].

The calibration of the proposed device was performed with experimental data [17] as shown in Figure 2. A device with a 100 nm gate length is calibrated with experimental work of heterojunction-free GaN FinFET [17] for more validation with our simulated data. This well calibrated result (Figure 2) shows the validity of simulation models. 

## 4. Result and Discussion

**Figure 3:**
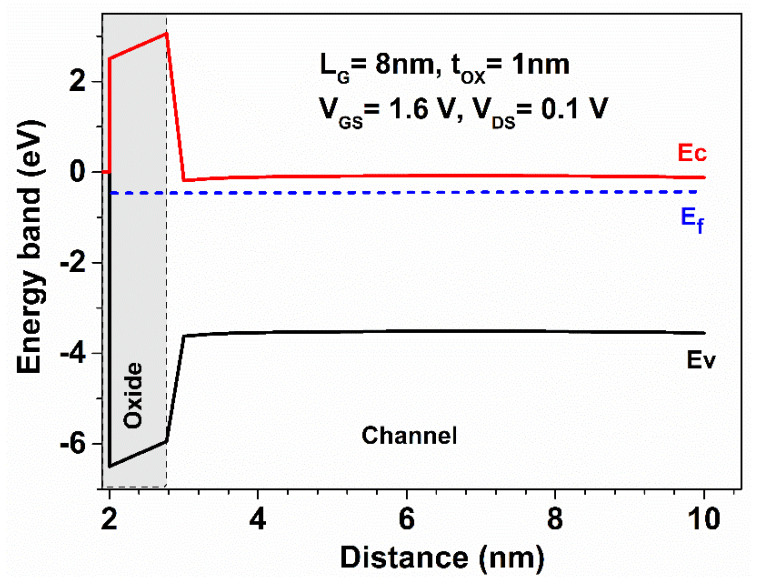
Energy Band diagram for the vertical cross-section of GaN-SOI FinFET.

**Figure 4:**
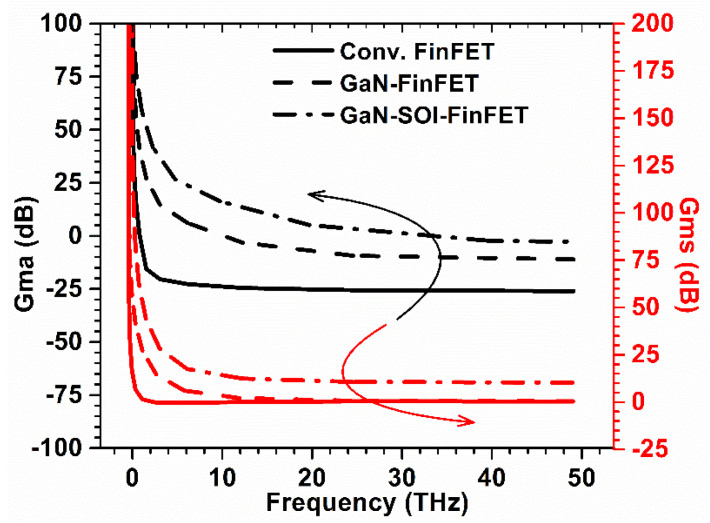
Gma and Gms for Conventional and GaN-SOI-FinFET.

The energy band diagram of the proposed GaN-SOI-FinFET device across the vertical cross-section (with conduction band, valence band, and Fermi level) is depicted in Figure 3. For the improved RF performance of the proposed device, various gain parameters were evaluated. In order to evaluate power gains, first Gma and Gms were evaluated for conventional FinFET, GaN-FinFET, and GaN-SOI-FinFET using Equation (1) [18]. All the data for Gma and Gms were plotted against frequency (in THz range) as reflected in Figure 4. Figure 4 shows the improved performance of GaN-SOI-FinFET in comparison with GaN-FinFET and conventional FinFET. Gma and Gms represent the desirable maximum theoretical power gain of the device. To define the Gma, a two-port network was considered and defined as the ratio of the power available (maximum) at load to the power available (maximum) at the source. GaN-SOI-FinFET depicts substantial improvement in Gma and Gms due to the electrical properties of GaN as the larger value of Gma and Gms is desirable for high-frequency applications.

(1) $$G_{ma}=\frac{P_{load,\max}}{P_{source,\max}}$$

Low-noise amplifier (LNA) designing is as important as power gains for RF amplifiers and in this way stability is the key parameter. An amplifier’s stability is also known as the Stern stability factor (K), and has a value of K usually >1 at high frequency and <1 at low frequency [19,20]. K can be defined as in Equation (2) [18,21],

(2) $$K=\frac{1+{\left|\Delta \right|}^{2}-{\left|S_{11}\right|}^{2}-{\left|S_{22}\right|}^{2}}{2.\left|S_{12}\cdot S_{21}\right|}$$

Here, coefficients of reflection are denoted by *S*_11_ and *S*_22_, while coefficients of transmission are denoted by *S*_12_ and *S*_21_, and Δ is represented as

(3) $$\Delta =S_{11}\times S_{22}-S_{12}\times S_{21}$$

**Figure 5:**
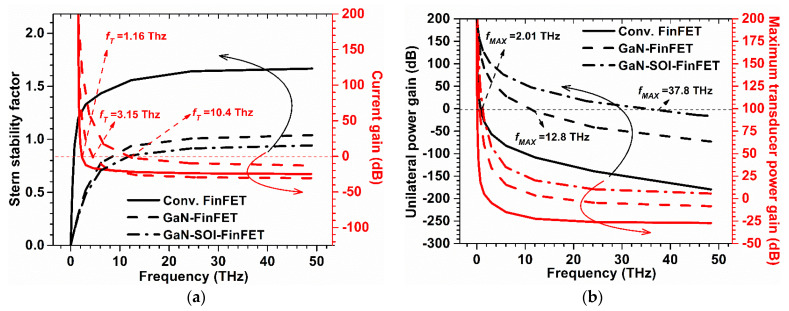
(**a**) SS and Current Gain for Conventional and GaN-SOI-FinFET. (**b**) Unilateral Power Gain and G_MT_ for Conventional and GaN-SOI-FinFET.

The Stern stability factor for both the devices is shown in Figure 5. From Figure 5, it can be observed that K is greater than one at high frequency for the GaN-FinFET and conventional FinFET, and ~1 (slightly > 1) for the proposed device at a higher frequency; this is desirable for the designing of RF amplifiers.

**Figure 6:**
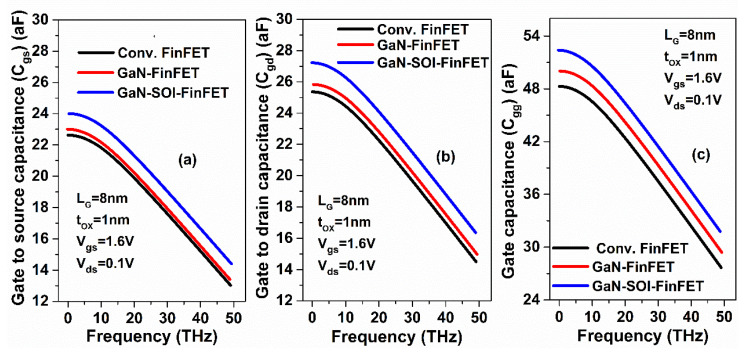
Parasitic capacitances (**a**) C_gs_, (**b**) C_gd_, and (**c**) C_gg_; plots against high frequencies (THz) for Conv. FinFET and GaN-SOI-FinFET.

**Figure 7:**
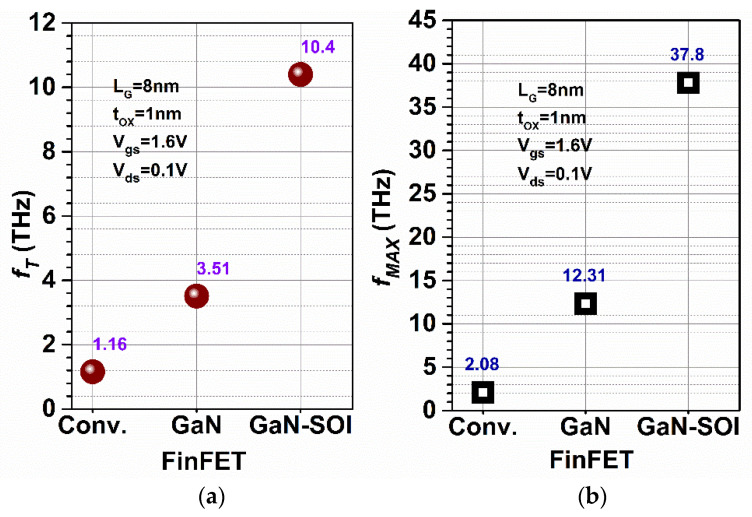
(**a**) *f*_T_ and (**b**) *f*_MAX_ for Conventional and GaN-SOI-FinFET.

**Figure 8:**
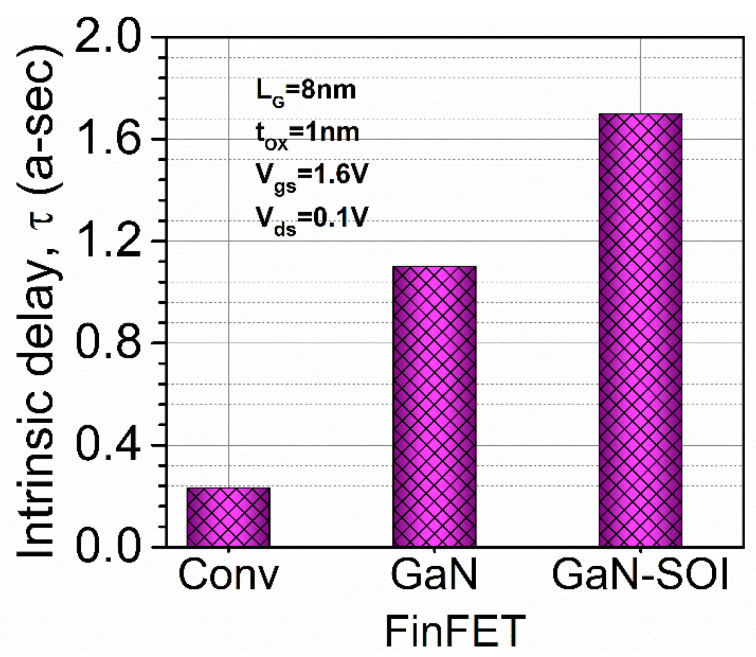
Intrinsic delay for Conv. FinFET and GaN-SOI-FinFET.

Further, cut-off frequency (*f*_T_) was extracted from the current gain plot at unity current gain [22] (shown in Figure 5a). *f*_T_ can be defined by Equation (4) and it measures the swing and speed of high-speed digital applications [23,24,25]:

(4) $$f_{T}=\frac{g_{m}}{2\pi \left(C_{gs}+C_{gd}\right)}$$

where *g_m_* is the transconductance and stray capacitances are represented by the gate to source capacitance (*C_gs_*) and gate to drain capacitance (*C_gd_*). These stray (parasitic) capacitances were also evaluated and plotted against frequency as shown in Figure 6. The results show that *f*_T_ increases nine-fold in GaN-SOI-FinFET compared to conventional FinFET and two-fold compared to GaN-FinFET, as shown in Figure 7a, due to reduced values of capacitances (shown in Figure 6). The maximum oscillator frequency (*f*_MAX_) was also evaluated at the unity unilateral power gain [26] (shown in Figure 5b). *f*_MAX_ should be as high as possible for RF applications and can be calculated as in Equation (5) [22,27]:

(5) $$f_{MAX}=\frac{f_{T}}{\sqrt{4R_{g}\left(g_{ds}+2\pi f_{T}C_{gd}\right)}}$$

where *g_ds_* is drain conductance and *R_g_* is gate resistance. Figure 5b shows that *f*_MAX_ enhances ten-fold and two-fold in the GaN-SOI-based device as compared to GaN-FinFET and silicon-based FinFET, respectively, as clearly reflected in Figure 7b. Figure 5 also shows the RF performance of the device and the efficacy of the two ports in terms of *G_MT_*, which is an evaluation of the effectiveness of the two ports. *G_MT_* is improved in the GaN-based device compared to GaN-FinFET and silicon-based FinFET, as shown in Figure 5b, owing to GaN material in the channel region which made the device more suitable for RF applications. Thereafter, intrinsic delay (as shown in Figure 8) was calculated and plotted for both conventional FinFET and GaN-SOI-FinFET.

(6) $$\tau =\frac{C_{gg}\cdot V_{d}}{I_{ON}}$$

where applied drain bias voltage is denoted by *V_d_, C_gg_* is the gate capacitance evaluated and plotted in Figure 6, and I_ON_ is the on-current of the device. Figure 8 reflects a very high reduction (85.29%) in intrinsic delay in the GaN-based device compared to the silicon-based FinFET device due to the reduction in gate capacitances and higher current driving capability. Thus, GaN-SOI-FinFET proves to be the most suitable candidate for RF applications.

**Figure 9:**
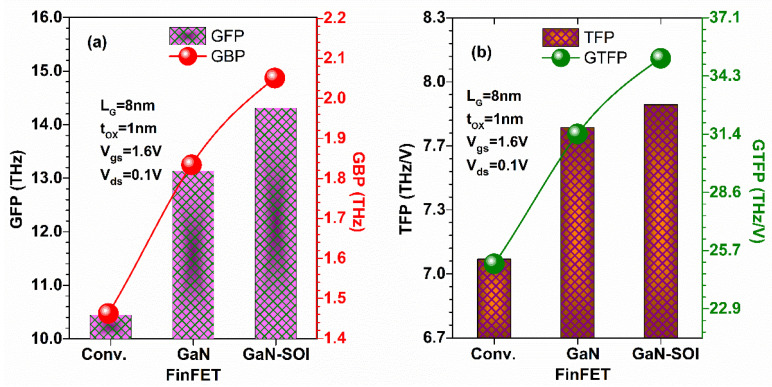
(**a**) GFP and GBP for GaN-SOI, GaN, and Conv. FinFETs. (**b**) TFP and GTFP for GaN-SOI, GaN, and Conv. FinFETs.

For high-frequency applications, GFP (gain frequency product) is one essential parameter and is given in Equation (7). Figure 9a shows that GFP is enhanced in the proposed device compared to the other two devices due to the higher value of *f*_T_ and hence the gain bandwidth product (GBP) increases. Moreover, other important RF parameters were evaluated in terms of gain transconductance frequency product (GTFP) and transconductance frequency product (TFP), as expressed in Equations (8) and (9), respectively. TFP is utilized in high-speed designs as it exhibits an agreement between bandwidth and power [28]. Figure 9b shows the TFP and GTFP for GaN-SOI, GaN, and Conv. FinFETs. The result reflects that both TFP and GTFP increase for Gan-SOI-FinFET compared to GaN and Conv. FinFETs and then attain a maximum value due to the higher value of *f*_T_. Thus, GaN-SOI-FinFET is the most suitable device design in terms of power gain improvements for RF applications.

(7) $$GFP=\left(\frac{g_{m}}{g_{d}}\right)\times f_{T}$$

(8) $$GTFP=\left(\frac{g_{m}}{g_{d}}\right)\times \left(\frac{g_{m}}{I_{d}}\right)\times f_{T}$$

(9) $$TFP=\left(\frac{g_{m}}{I_{d}}\right)\times f_{T}$$

### 4.1. Impact of Gate Length Variation

**Figure 10:**
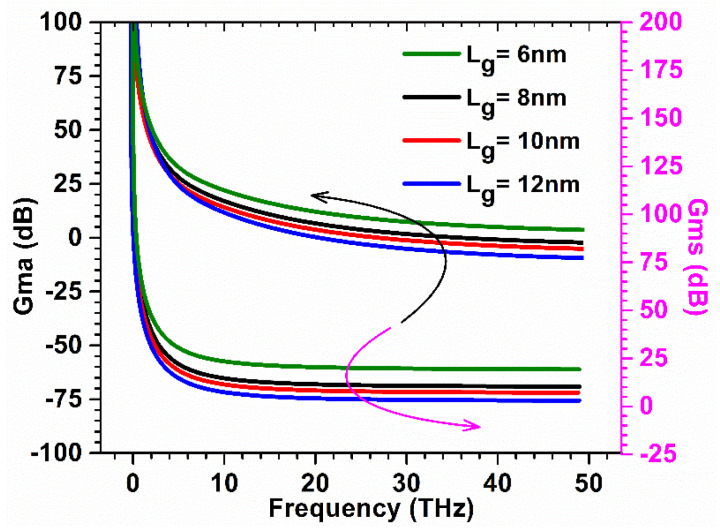
Gma and Gms at various gate lengths of GaN-SOI-FinFET.

Further, to optimize the device parameters, the improved RF performance gate length was varied. Power gain parameters Gma and Gms were evaluated for GaN-SOI-FinFET for various gate lengths using Equation (1) [18]. All the data for Gma and Gms were plotted against frequency (in THz range), as reflected in Figure 10, for various gate lengths. Figure 10 shows that when the gate length is reduced from 12 nm to 6 nm, the gains are improved. GaN-SOI-FinFET depicts substantial improvement in Gma and Gms at 6 nm gate length, which is desirable for high-frequency applications.

**Figure 11:**
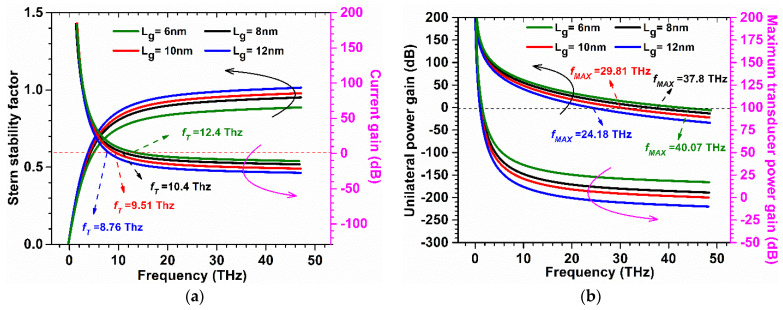
(**a**) SS and Current Gain at various gate lengths of GaN-SOI-FinFET. (**b**) Unilateral Power Gain and G_MT_ at various gate lengths of GaN-SOI-FinFET.

**Figure 12:**
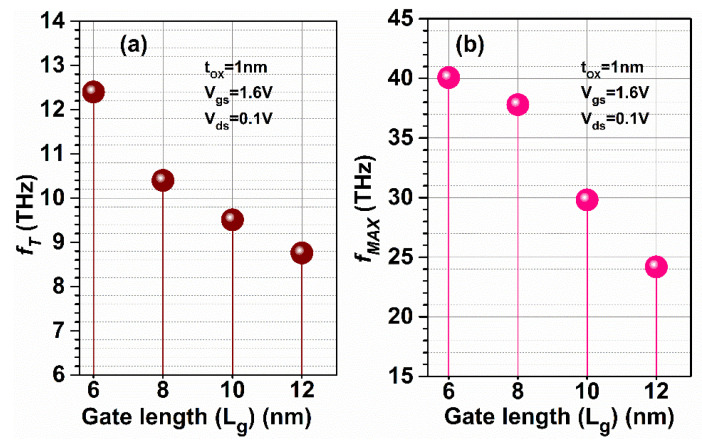
(**a**) *f*_T_ and (**b**) *f*_MAX_ at various gate lengths of GaN-SOI-FinFET.

The Stern stability factor was also observed for different gate lengths as a function of frequency for GaN-SOI-FinFET, as shown in Figure 11a. From Figure 11a, it was observed that the Stern stability factor improved (slightly < 1) for reduced gate length (6 nm) in the proposed device at a higher frequency and it is desirable for the designing of RF amplifiers. *f*_T_ was extracted from the current gain plot at unity current gain (shown in Figure 11a) for various gate lengths at very high frequencies. When gate length is reduced from 12 nm to 6 nm, *f*_T_ increases by 28% due to a reduction in stray capacitances, as clearly reflected in Figure 12a. 

**Figure 13:**
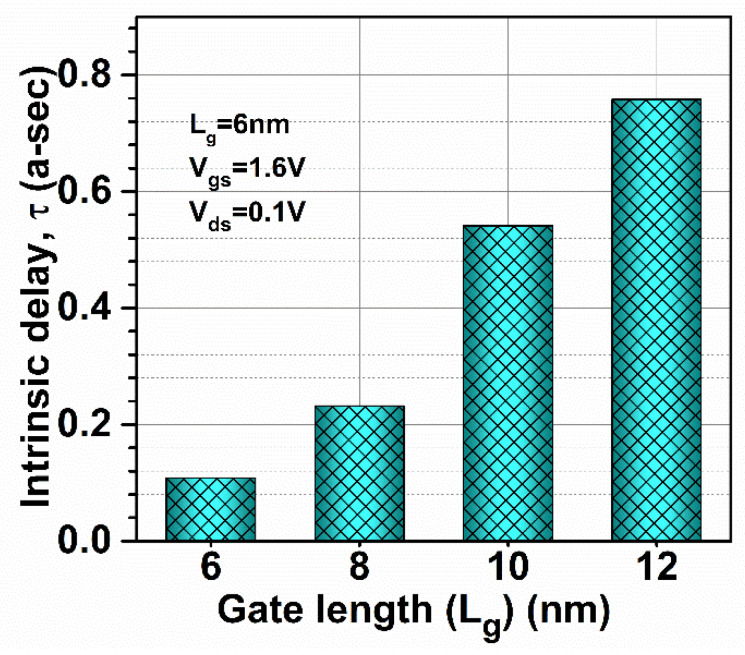
Intrinsic delay at various gate lengths of GaN-SOI-FinFET.

*f*_MAX_ was also evaluated at the unity unilateral power gain for different gate lengths (shown in Figure 11b). Figure 11b shows that *f*_MAX_ is enhanced by 66.66% when gate length is reduced, as shown in Figure 12b. Figure 11b also shows the RF performance of the device in terms of G_MT_ for different gate lengths and it is observed that G_MT_ is improved with the reduction in gate length to 6 nm in the proposed device, which makes the device more suitable for RF applications at 6 nm gate length. Thereafter, intrinsic delay (as shown in Figure 13) was calculated and plotted for varied gate lengths. Figure 13 reflects a very high reduction (86.66%) in intrinsic delay when gate length is reduced from 12 nm to 6 nm due to the reduction in gate capacitances and higher current-driving capability. Thus, GaN-SOI-FinFET with a 6 nm gate length proves to be the most suitable candidate for RF applications.

**Figure 14:**
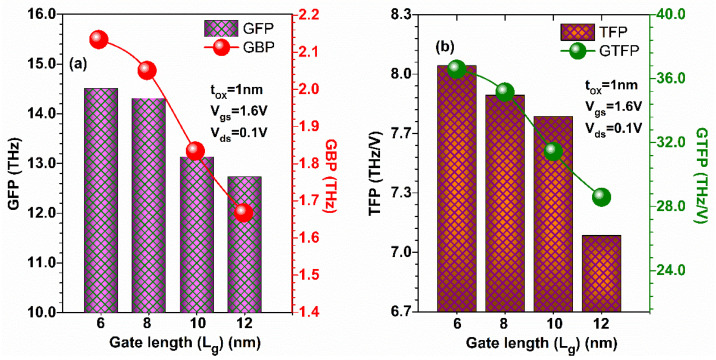
(**a**) GFP and GBP at various gate lengths of GaN-SOI-FinFET. (**b**) TFP and GTFP at various gate lengths of GaN-SOI-FinFET.

Figure 14a shows that GFP is enhanced (by 13.2%) in the proposed device when the gate length is reduced from 12 nm to 6 nm due to the higher value of *f*_T_ at a lower gate length and hence GBP increases by 29.69%. Moreover, GTFP and TFP were evaluated at different gate lengths, as shown in Figure 14b. Figure 14b shows TFP and GTFP increased in GaN-SOI-FinFET at lower gate lengths compared to longer gate lengths by 14.08% and 24.13%, respectively, and then attained a maximum value due to the higher value of *f*_T_. Thus, GaN-SOI-FinFET reflects more favourable results in terms of power gain improvements for RF applications at 6 nm gate length.

### 4.2. Impact of Oxide Thickness Variation

**Figure 15:**
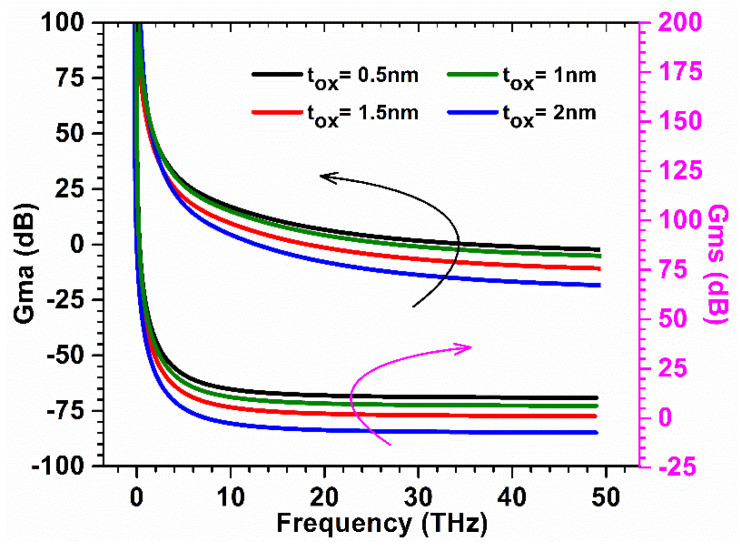
Gma and Gms at various oxide thicknesses of GaN-SOI-FinFET.

Moreover, oxide thickness was considered for the optimization of the device parameters to improve RF performance. Once again, we started with well known power gain parameters, Gma and Gms, which were evaluated for the proposed device for various oxide thicknesses. Both parameters were calculated and plotted against a frequency range of several THz for various oxide thicknesses, as reflected in Figure 15. Figure 15 shows that when oxide thicknesses are reduced from 1 nm to 0.5 nm, the gains are reduced due to gate leakage current and when oxide thicknesses increase from 1 nm to 2 nm, again the gains are reduced due to increased capacitances. GaN-SOI-FinFET depicts substantial improvement in Gma and Gms at 1 nm oxide thickness, which is the optimal value for high-frequency applications.

**Figure 16:**
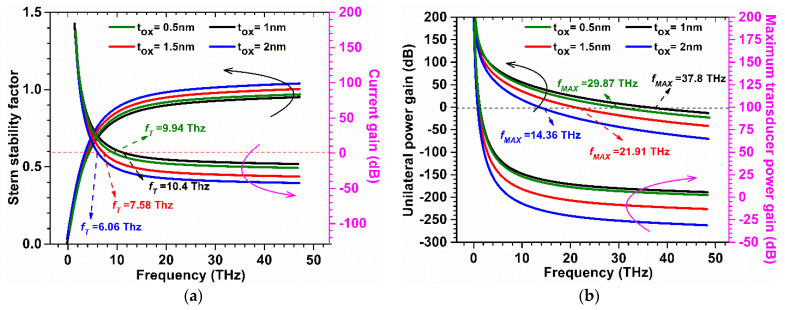
(**a**) SS and Current Gain at various oxide thicknesses of GaN-SOI-FinFET. (**b**) Unilateral Power Gain and G_MT_ at various oxide thicknesses of GaN-SOI-FinFET.

**Figure 17:**
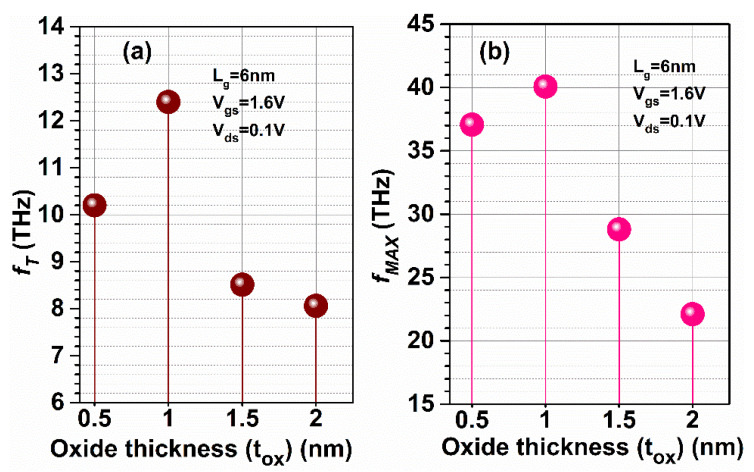
(**a**) *f*_T_ and (**b**) *f*_MAX_ at various oxide thicknesses of GaN-SOI-FinFET.

**Figure 18:**
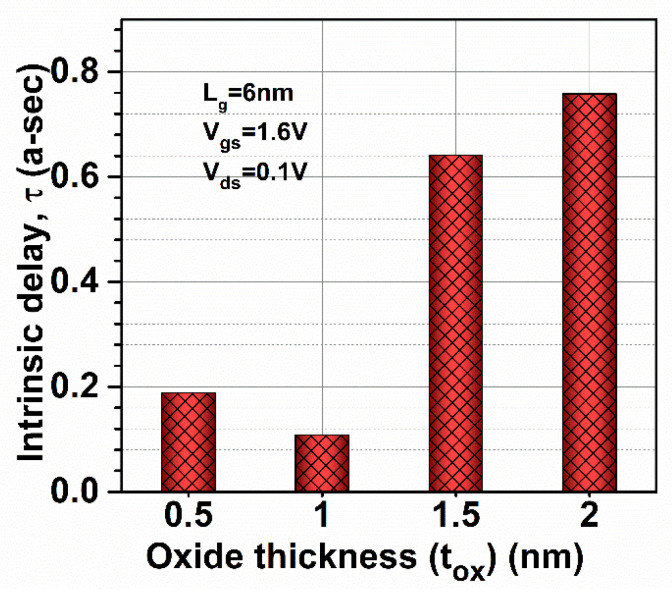
Intrinsic delay at various oxide thicknesses of GaN-SOI-FinFET.

The Stern stability factor was also observed to optimize the oxide thickness as a function of frequency (in the THz range) for GaN-SOI-FinFET, as shown in Figure 16a. From Figure 16a, it was observed that the Stern stability factor increases (slightly > 1) for reduced oxide thickness (0.5 nm), while increasing more when the oxide thickness increases to 2 nm at a higher frequency. For different oxide thicknesses at very high frequencies, *f*_T_ was extracted from the current gain plot at unity current gain, as shown in Figure 16a. When oxide thickness is reduced from 1 nm to 0.5 nm, *f*_T_ is slightly reduced (by 20.31%); however, more reduction is observed when oxide thickness increases to 2 nm (by 37.5%) due to increased stray capacitances, as clearly reflected in Figure 17a. *f*_MAX_ was also evaluated at the unity unilateral power gain for different gate lengths, as shown in Figure 16b. Figure 16b shows that *f*_MAX_ is slightly reduced (by 7.5%) when oxide thickness is reduced from 1 nm to 0.5 nm; however, more reduction is observed when oxide thickness increases to 2 nm (by 45%) due reduction in *f*_T_ as shown in Figure 17b. Figure 16b also shows the RF performance of the device in terms of G_MT_ for different oxide thicknesses and it is observed that G_MT_ is slightly reduced when oxide thickness is reduced from 1 nm to 0.5 nm; however, more reduction is observed when oxide thickness increases to 2 nm. Thereafter, the intrinsic delay was calculated and plotted for various oxide thicknesses, as shown in Figure 18. Figure 18 reflects increased values (by 98%) in intrinsic delay when oxide thickness is reduced from 1 nm to 0.5 nm; however, a higher value (by 6.5 times) is observed when oxide thickness increases to 2 nm due to reduction in gate capacitances. Thus, GaN-SOI-FinFET with 1 nm oxide thickness proves to be the most suitable candidate for RF applications.

**Figure 19:**
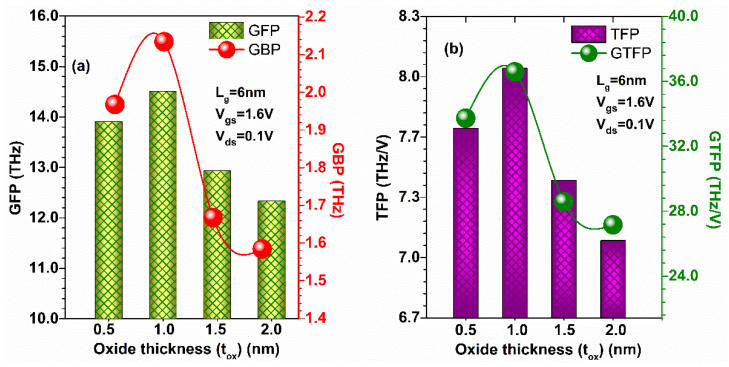
(**a**) GFP and GBP at various oxide thicknesses of GaN-SOI-FinFET. (**b**) TFP and GTFP at various oxide thicknesses of GaN-SOI-FinFET.

Figure 19a shows that GFP is slightly reduced (by 4.82%) when oxide thickness is reduced from 1 nm to 0.5 nm; however, more reduction is observed when oxide thickness increases to 2 nm (by 15.86%) due to the reduction in *f*_T_ compared to 1 nm oxide thickness, and hence GBP also follows the same patterns (by 9.3% and 26.5%). Thereafter, GTFP and TFP were evaluated at different oxide thicknesses, as shown in Figure 19b. Figure 19b shows the TFP and GTFP show the higher value at 1 nm oxide thickness and reduce their values when oxide thickness decreases/increases to 0.5/2 nm due to the change in *f*_T_. Thus, GaN-SOI-FinFET reflects more favourable results in terms of power gain improvements for RF applications at 1 nm oxide thickness.

## 5. Conclusions

The presented work investigated the efficacy of the GaN-based SOI-FinFET device for improved RF performance. From the results, it was observed that GaN-SOI-FinFET enhanced gain matrices significantly in terms of Gma, Gms, and G_MT_. The capacitances (parasitic) were also measured in terms of *C_gs_*, *C_gd_*, and *C_gg_* and it is observed that these capacitances are reduced significantly, which enhances the cut-off frequency by ~9 times and the *f*_MAX_ by ~18 times in the GaN-based SOI-FinFET compared to Conv. FinFET and GaN-FinFET. The intrinsic delay was also reduced by 6.8 times in GaN-based SOI-FinFET compared to silicon-based FinFET. Further, to find the optimized value of gate length and oxide thickness, parametric assessment was also performed and it was found that 6 nm gate length and 1 nm oxide thickness are the optimized values for desired RF performances. Thus, all the results show that GaN-based SOI-FinFET is the most suitable solution for high-performance RF applications in the sub-10 nm regime.

## Figures and Tables

**Table 1:** Device Parameters.

Parameter	Dimension
L_G_ (nm)	8
Length of Source (L_S_) and Drain (L_D_) (nm)	10
Fin Height, H_Fin_ (nm)	8
Fin Width, W_Fin_ (nm)	4
Oxide Thickness, t_OX_ (nm)	1
N_Ch_ (cm^−3^)	1.0 × 10^16^
N_S,D_ (cm^−3^)	1.0 × 10^21^

## Data Availability

Data underlying the results presented in this paper are not publicly available at this time but may be obtained from the authors upon reasonable request.
